# Effect of Preweaning Socialization on Postweaning Biomarkers of Stress, Inflammation, Immunity and Metabolism in Saliva and Serum of Iberian Piglets

**DOI:** 10.3390/ani16010088

**Published:** 2025-12-28

**Authors:** Carolina Becerra, Francisco Ignacio Hernández-García, Antonia Gómez-Quintana, José Joaquín Cerón, María Botía, Clara Mateos, Mercedes Izquierdo

**Affiliations:** 1 Center of Scientific and Technological Research of Extremadura (CICYTEX), 06187 Guadajira, Spain; carolina.becerra@juntaex.es (C.B.); francisco.ignacio.hernandez@gmail.com (F.I.H.-G.); antonia.gomezq@juntaex.es (A.G.-Q.); claramaria.mateos@juntaex.es (C.M.); 2 Salilab-UMU, Interdisciplinary Laboratory of Clinical Analysis (Interlab), Regional Campus of International Excellence “Campus Mare Nostrum”, University of Murcia, 30100 Murcia, Spain; jjceron@um.es (J.J.C.); maria.botiag@um.es (M.B.)

**Keywords:** Iberian piglets, socialization, stress, biomarkers

## Abstract

Weaning is one of the most stressful stages in pig production, especially for Iberian piglets, which grow more slowly than other cosmopolitan breeds and therefore, have a lower weaning weight when raised in intensive systems. Stress at weaning, caused by separation from the sow, dietary change, and regrouping with unfamiliar piglets, can negatively impact welfare, immune function, and performance. Pre-weaning socialization, which allows piglets from different litters to interact before weaning, has been proposed as a strategy to reduce aggression and facilitate the adaptation to the post-weaning period. However, its physiological effects in Iberian pigs remain largely unknown. In this study, 8 Iberian sows and their litters were assigned to either a control group (CTRL) or a socialization group (SOC) where litters were mingled (socialized) two weeks before weaning. Salivary and serum biomarkers of stress, inflammation, immunity and metabolism were measured at weaning (pwD0) and 7 days post-weaning (pwD7), and growth performance was recorded until 60 days of age. Socialized piglets showed reduced salivary Adenosine Deaminase (ADA) activity at pwD0 and pwD7 and lower salivary chromogranin A (CgA) and serum Haptoglobin (Hp) levels at pwD7. In contrast, they presented higher concentrations in serum of high-density lipoprotein (HDL), low-density lipoprotein (LDL), total cholesterol, glucose, and urea at pwD7 compared to controls. Attending to the sex effect, Butyryl-cholinesterase (BChE) serum concentration was higher in males and urea, and creatinine were higher in females. Growth rates were higher in socialized piglets in the first two weeks post-weaning but lower thereafter. These findings may suggest that pre-weaning socialization could reduce the stress associated with early post-weaning in Iberian piglets, thus potentially improving welfare and adaptation during this period.

## Simple Summary

Weaning is a challenging stage for piglets, especially for those of the Iberian breed, which grow more slowly than piglets from other cosmopolitan breeds and therefore, have lower weaning weights when raised in intensive systems. The abrupt separation from the sow, the dietary change, and the mixing with unfamiliar piglets can cause stress, affecting welfare, immunity, and growth. In this study, Iberian piglets were either kept in their litter groups or allowed to mix with other litters from 15 days of age, a process known as pre-weaning socialization. We measured different indicators of stress, inflammation, immunity and metabolism in saliva and blood at weaning and 7 days post-weaning. Socialized piglets showed signs of reduced stress and inflammation after weaning, along with changes in metabolic indicators, compared to non-socialized piglets. Although they grew faster in the first two weeks post-weaning, their growth slowed later. These results suggest that pre-weaning socialization may help Iberian piglets adapt better to weaning, improving their welfare and physiological homeostasis.

## 1. Introduction

Weaning constitutes one of the most stressful periods in a piglet’s life, especially in intensive management systems and breeds such as the Iberian pig (a rustic and fatty breed from the southwestern regions of the Iberian Peninsula).

Stress at weaning caused by the separation from the sow, dietary change from milk to solid feed, relocation to new environments, and regrouping with unfamiliar piglets act as potent stressors [1]. These stressful conditions can activate the hypothalamic–pituitary–adrenal (HPA) axis, that releases glucocorticoids to mobilize protein and fat reserves, and the sympatho–adreno–medullary (SAM) system, that stimulates catecholamine release to increase heart rate, blood pressure and energy availability [2], triggering physiological responses aimed at coping with the stressor but which can also impair piglets’ homeostasis [3], resulting in an alteration of welfare and performance.

One factor closely linked to post-weaning stress is the social reorganization that occurs when piglets are mixed with non-littermates, subsequent aggressive interactions to establish a new dominant hierarchy can lead to physical injuries, reduced feed intake, and elevated stress biomarkers [3]. Pre-weaning socialization, defined as the controlled mingling of litters before weaning, has been proposed as a management strategy to reduce the severity of these aggressive encounters [4,5]. By allowing piglets to interact and familiarize themselves with non-littermates before weaning, socialization may enhance social skills, reduce post-weaning stress, and promote better physiological adaptation to that period.

Considering that Iberian pigs belong to a breed traditionally raised in extensive systems (Mediterranean cleared forest), weaned at a later age than intensive breeds and with a highly adipogenic metabolism [6,7], it could be expected that their response to stress at weaning in intensive systems at earlier ages and lower weights would be different from that of fast-growing breeds, as likewise, a different response could result from pre-weaning socialization.

Research in commercial breeds has reported that pre-weaning socialization can improve behavioral outcomes, including reduced fighting duration and lower injury scores after regrouping, as well as potential benefits in early growth and physiological stress responses [4,5]. However, results are not always consistent and vary between authors, possibly due to increased early-life competition or stress during the socialization phase itself [8,9], or by the different methodologies applied. Moreover, most of this research has been conducted in fast-growing breeds, and the specific physiological responses of Iberian pigs to early socialization remain largely unexplored.

Stress responses can be assessed through various biomarkers in saliva and blood, reflecting activation of neuroendocrine, immune, and metabolic pathways [10]. Salivary cortisol (Cort) and cortisone (Crts) are widely used indicators of HPA axis activity, while chromogranin A (CgA) and alpha amylase (sAA) reflect SAM system activation. Other salivary analytes such as oxytocin (OT) may be linked to social bonding and emotional regulation, whereas adenosine deaminase (ADA) activity can reflect immune system activation [11]. In serum, acute-phase proteins such as haptoglobin (Hp) and C-reactive protein (CRP) are key indicators of systemic inflammation [12], while metabolic parameters (e.g., glucose, lipids, urea) provide insight into energy balance and nutrient utilization. The assessment of these multiple biomarkers may contribute to understanding how pre-weaning socialization affects different physiological systems, as well as the mechanisms underlying behavioral and productive responses that could not be detected by single-marker approaches. Furthermore, this multi-marker approach may help to evaluate pre-weaning socialization as a possible management strategy to improve welfare and productivity.

As far as we know, there are no studies addressing simultaneous changes in such a variety of biomarkers in response to different aspects of stress at weaning, either in Iberian pigs or in other swine breeds. In addition, given the importance of social behavior in piglets, especially around weaning, and its possible modifications by management strategies at early stages, such as the pre-weaning period, the objectives of the present study were the following: (1) To investigate the effects of pre-weaning socialization on growth performance and biomarkers related to stress, inflammation and immunity in Iberian piglets. (2) To characterize the neuroendocrine, inflammatory, immune and metabolic responses to stress at weaning and during early-postweaning in Iberian piglets through salivary (non-invasive and easy to collect) and serum biomarkers. (3) To provide new information, based on a multi-biomarker approach, on several still poorly known stress-indicators which may enable useful comparisons and be important for similar future studies.

## 2. Materials and Methods

The present study was conducted at the CICYTEX facilities in Southwestern Spain, “Finca Valdesequera” (coordinates 39°3′13.2″, −6°50′45.2″) during the cold season (winter) of 2024–2025. All experimental procedures complied with the European Union regulations on the protection of animals used for scientific and experimental purposes (Directive 2010/63/EU), ensuring that ethical standards were followed throughout the research process to minimize any potential distress or harm to the animals involved. The research protocols were reviewed and approved by the Ethics Committee of the University of Extremadura, Spain, under reference number 174/2024.

### 2.1. Animals and Experimental Design

A total of 8 litters of the Iberian pig breed, having 6 or more piglets each, were chosen to carry out the experiment. Litters were randomly assigned to one of the two treatment groups: Control (CTRL, n = 4 litters) and Socialized (SOC, n = 4 litters). In the latter, piglets were allowed to socialize among litters starting at 15 days of age. These 8 litters had a total of 64 male and female Iberian piglets (CTRL, n = 32 piglets; SOC, n = 32 piglets). During the pre-weaning phase, piglets remained with their dams in cubicles (2.40 × 1.60 m) with farrowing crates in a slatted-floor nursing facility with regulated temperature and humidity (22 ± 0.5 °C and 70 ± 5%, respectively). In addition to the sow crate, each cubicle had a heating plate (30 ± 2 °C), a nipple drinker and a creep-feed floor dish for the piglets.

On their day 15 of life (lactation day 15; LD15) piglets were introduced to the creep feed. Also on this day, the socialization process began in the SOC group with the removal of the separation panels of four farrowing cubicles. Specifically, the four litters assigned to the SOC treatment were housed in adjacent cubicles, and all partition panels between these cubicles were removed simultaneously on LD15, creating a single common space (approximately 9.6 × 1.6 m) where all piglets from the four litters could freely interact with each other and have access to the four sows. This arrangement allowed for complete social mixing among the four socialized litters from LD15 until weaning.

At the time of weaning (30 ± 2 days of life), from the 64 piglets, a total of 46 male and female were selected excluding piglets that weighed more than ± one standard deviations from the mean, remaining only the central piglets, thus reducing unwanted weight variation. These 46 piglets were assigned to pens according to litter and sex to continue the experiment. Although 8 litters, 4 per treatment is not a large sample, the selection of the 46 piglets was performed to maintain balanced representation across litters and sex within each treatment group. Specifically, from each of the 4 litters assigned to each treatment, 5–6 piglets were selected (2–3 males and 2–3 females per litter), ensuring that the sex ratio was balanced within each treatment group. These postweaning groups were the following: CTRL (n = 13 males and 10 females) and SOC (n = 13 males and 10 females), which corresponded to the preweaning treatment for each piglet. At this moment, the animals were transferred to pens (5 to 7 piglets/pen) with slatted floor provided with heating plates (30 ± 2 °C), nipple drinkers and feeding hoppers. Piglets from different litters were mixed in the post-weaning pens for both treatment groups. Each pen contained piglets from 3 to 4 different litters, with a similar litter mixing rate applied to both CTRL and SOC groups, thus reducing the mother effect. During the postweaning period, which was monitored until day 30 ± 2 post-weaning (D60 of age), piglets were fed ad libitum with the same commercial pre-starter feedstuff that they were given during the pre-weaning period. This concentrate feed was free of antibiotics and ZnO (see Table 1).

### 2.2. Data and Sample Collection

#### 2.2.1. Saliva Sampling

Saliva samples were collected at weaning (pwD0) and 7 days post-weaning (pwD7) at the same time of the day (10 a.m.) to avoid sampling during the peak cortisol response that takes place early in the morning [13]. This timing was chosen to minimize the confounding effects of circadian variation in stress hormones, particularly cortisol and cortisone, which exhibit diurnal rhythms in pigs [13]. Saliva samples were collected approximately one and a half hours after the piglets were transferred to the post-weaning pens, allowing time for the acute stress of handling and relocation to subside while still being under the influence of the stress response associated with maternal separation and environmental change, and also under the stress of the possible hierarchical fights that usually occur after group mixing when the piglets came from litters without previous social contact. Samples were collected using commercial collection kit tubes (Salivette^®^, Starstedt, Germany). Collection time was 2 ± 0.5 min per piglet to allow the absorption of an adequate amount of saliva. The procedure involved inserting a polyurethane sponge (2 × 1.5 × 1.5 cm) attached to the end of a reusable collection device specially designed for this study, consisting of a metal rod with a plastic protective cover into the piglet’s mouth.

After collecting, samples were immediately refrigerated until transported to the laboratory. They were then centrifuged at 16 °C, 4000 rpm for 10 min (Eppendorf Centrifuge 5810, Hamburg, Germany). The supernatant was collected from the kit tube and transferred into 1.5 mL Eppendorf tubes, which were stored at −80 °C until further analyses.

#### 2.2.2. Blood Samples

Blood samples were obtained after saliva collection using 4.5 mL vacuum tubes on pwD7 by applying the cranial vena cava puncture method. Samples were obtained from all piglets following standard veterinary procedures by an experienced veterinarian and the procedure time was limited to a maximum of 2 min per piglet, to ensure animal welfare. To minimize piglet stress and optimize puncture precision, the animal was securely immobilized in the supine position by manually holding its limbs and head while resting its back on the midline of a 50 × 60 cm, V-shaped (dihedral) support. The vacuum tubes, which had a clot activator for serum obtention, were kept refrigerated and then centrifuged at 21 °C, 2000 rpm for 10 min. The supernatant serum was then collected and transferred into 5 mL plastic test tubes and stored at −80 °C until further analyses.

#### 2.2.3. Growth Measurements

Individual pig BW was recorded every 15 days, from birth to D60 of age: LD0 (lactation day 0, at birth), LD15 (lactation day 15, D15 of age), LD30 (pwD0; weaning, D30 of age), pwD15 (post-weaning day 15, D45 of age) and pwD30 (post-weaning day 30, D60 of age). To be consistent, each day the weight sampling was carried out at the same time. Average daily gain (ADG; kg/day) was calculated for selected intervals between these time points by dividing the BW difference by the number of days in each interval.

### 2.3. Biomarker Analyses

#### 2.3.1. Salivary Biomarker Analyses

**Figure 1:**
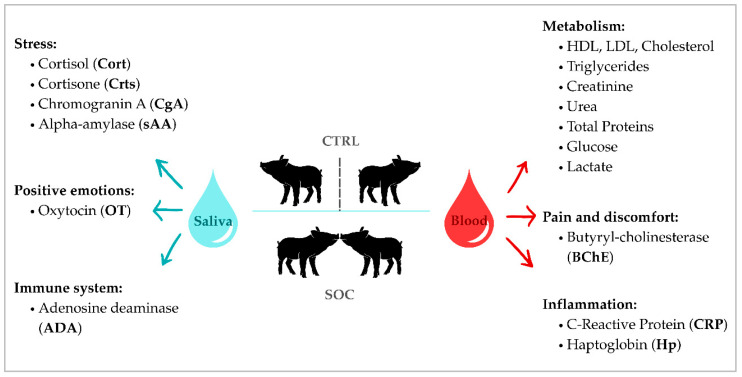
Summary of the different biomarkers analyzed in saliva and blood for Control (CTRL) and Socialized (SOC) piglets at weaning and on day 7 post-weaning.

Salivary biomarkers are summarized in Figure 1 diagram. The cortisol concentration was determined using a competitive enzyme immunoassay with chemiluminescence on a solid phase (IMMULITE 1000, Siemens Medical Solutions Diagnostics, Los Angeles, CA, USA) adapted for pig saliva. Results are presented in µg/dL. The activity of ADA and sAA was determined by an automated colorimetric assay using commercial kits (Diazyme Laboratories, Poway, CA, USA), results expressed in U/L. Oxytocin, CgA and cortisone were analyzed using AlphaLISA technology described by Botía et al. [14] (PerkinElmer, Inc., Waltham, MA, USA). Concentrations measured in pg/mL for oxytocin and in ng/mL for CgA and cortisone.

#### 2.3.2. Serum Biomarker Analyses

Serum biomarkers are summarized in Figure 1 diagram. To determine the concentration of acute-phase proteins such as CRP and Hp, serum samples were analyzed using immunoturbidimetric assays with commercial reagents from SPINREACT, S.A.U (Girona, Spain) and Beckman Coulter^®^ (Brea, CA, USA), respectively, with values reported in mg/L for CRP and in g/L for haptoglobin. The concentration of immunoglobulin G (IgG) was determined by a commercial ELISA kit (Pig IgG ELISA Kit; Bethyl Laboratories Inc., Montgomery, AL, USA), and the results are shown in mg/L. The activity of paraoxonase 1 (PON-1) was measured by an automated assay using 4-(p)-nitrophenyl acetate as substrate and the results are expressed in U/L. Butyryl-cholinesterase (BChE) activity was analyzed following the assay validated by Tecles & Cerón [15], and the results are given in µmol mL/minute. Metabolic parameters such as high-density lipoprotein (HDL), low-density lipoprotein (LDL), total cholesterol, triglycerides, creatinine, urea, glucose, lactate and total proteins were determined with a compact multiparametric autoanalyzer for biochemical analysis (Olympus AU600 Automatic Chemistry Analyzer, Olympus Europe GmbH, Hamburg, Germany), and the results are displayed in g/L for the total proteins and in mg/dL for the remaining metabolites.

### 2.4. Statistical Analysis

The data were analyzed using SAS 9.4 (SAS 9.4 package). The sow line (Valdesequera line, from CICYTEX, Badajoz, Spain) is a closed and fairly homogeneous line; however, considering that the 46 piglets came from 8 sows, it would still be expected that some of the variance could be due to differences between mothers. For this reason, the piglets were selected to ensure that all mothers were evenly represented in the experiment, selecting no fewer than 5 offspring from each mother to minimize potential maternal effects and associated bias. For this reason, all metabolic parameters and weights were analyzed with the GLM (General Linear Model) procedure, including treatment (SOC vs. CTRL), sex (males vs. females), and the interaction between treatment×sex as independent variables. *p*-values below 0.05 were considered significant and *p*-values between 0.05 and 0.10 were considered to indicate statistical trends. No significant treatment × sex interactions were observed for any of the variables measured. Least square means (LSMEANS) and standard errors of each main effect, and *p*-values are presented in all tables.

## 3. Results

### 3.1. Salivary Biomarkers

At weaning (pwD0; Table 2), cortisol, cortisone, CgA and sAA concentrations showed no significant effects of treatment or sex. In contrast, oxytocin levels showed a trend to be higher in CTRL vs. SOC piglets, but there were no sex effects. Moreover, ADA levels were significantly higher in CTRL vs. SOC group. No significant differences were found for the rest of the variables.

On pwD7 (Table 2), as in the previous period, there was no effect of treatment or sex for cortisol concentrations. Oxytocin showed a trend to be higher for the SOC group, but no sex effect was found. The concentrations of CgA were lower in SOC compared to CTRL.

No significant differences were found for the other two biomarkers (sAA and ADA), although ADA showed a trend to be lower for the SOC group.

### 3.2. Serum Biomarker Results

#### 3.2.1. Stress and Inflammation Biomarkers

Biomarkers related to stress or inflammation responses are shown in Table 3. On pwD7, BChE activity did not differ significantly between treatments. However, a significant sex effect was observed, with females showing lower values than males. The levels of CRP, Paraoxonase-1 (PON-1), and IgG were not significantly affected by treatment or sex. Haptoglobin levels were significantly lower in SOC piglets compared to CTRL, but there were no sex effects.

#### 3.2.2. Metabolic Biomarkers

Table 4 depicts the concentration of blood serum metabolic parameters. The HDL, LDL, and total cholesterol levels were significantly higher in SOC piglets compared to CTRL. There was a trend for a sex effect for HDL levels, with females showing slightly higher values than males. Urea concentration was significantly affected by treatment and sex. Glucose levels were significantly higher in SOC animals with no effect of sex.

Other analytes such as triglycerides, creatinine, total proteins, and lactate showed no significant effects of treatment or sex. However, creatinine showed a trend for a sex effect, with females exhibiting higher values.

### 3.3. Growth Performance

**Figure 2:**
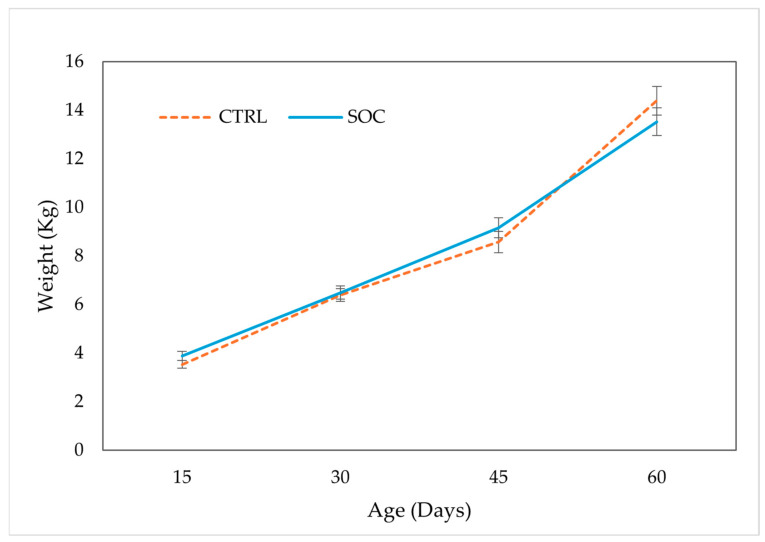
Body weight (LSMEAN ± SE) of piglets by treatment group (control, CTRL; socialized, SOC).

**Figure 3:**
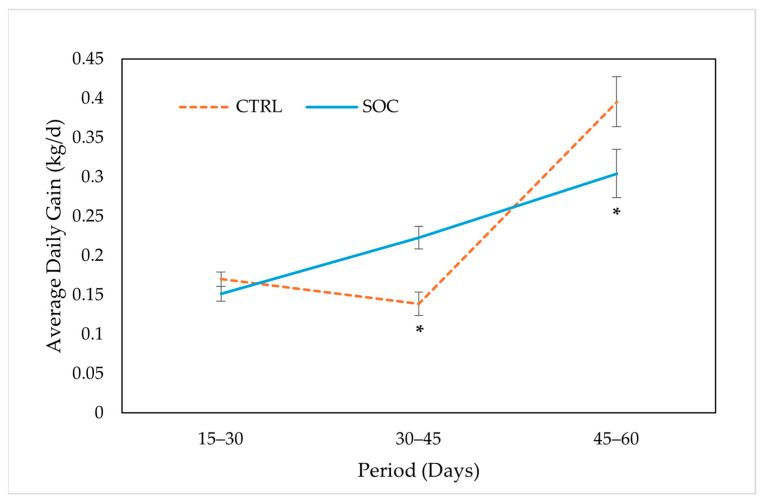
Average Daily Gain (LSMEAN ± SE) from days 15 to 60 of age by treatment group (control, CTRL; socialized, SOC. * indicates statistically significant differences (*p* < 0.05).). * Average Daily Gain (ADG) was measured in three intervals: from LD15 (lactation day 15) to LD30 (pwD0; weaning), from LD30 to day 45 of age, and from day 45 to day 60 of age.

Body weight (BW) data for all time points and Average Daily Gain (ADG) are presented in Figure 2 and Figure 3, respectively. No significant effects were found for BW at any time point for treatment (Figure 2) or sex. Regarding ADG, SOC piglets showed significantly higher growth rates than CTRL between D30 and D45 of age (0.22 vs. 0.14 kg/day, respectively). However, there was a significantly lower growth rate between D45 and D60 of age between treatments (0.30 vs. 0.40 kg/day, respectively). For the complete D30–D60 period, the ADG showed no significant difference between treatments and a trend for sex (CTRL= 0.26 ± 0.02 kg/d, SOC = 0.26 ± 0.02 kg/d; *p* = 0.935 and F = 0.24 ± 0.02 kg/d, M = 0.29 ± 0.02 kg/d; *p* = 0.07). No significant effects of sex were found for any of the other ADG intervals.

## 4. Discussion

### 4.1. Stress-Related Biomarkers

On weaning day, no significant differences were observed between SOC and CTRL piglets in salivary stress biomarkers such as cortisol or cortisone, key indicators of HPA axis activation.

Salivary cortisol and cortisone, although both derived from the HPA axis activation, may provide complementary information about stress responses. Cortisol is the primary glucocorticoid released during acute stress, whereas cortisone (the inactive metabolite of cortisol produced by 11β-hydroxysteroid dehydrogenase type 2) may reflect a more prolonged stress exposure and, in addition, it has been related to heat stress [14]. In our study, neither marker showed significant differences at weaning, suggesting that acute HPA axis activation was similar between treatments at this time point. Although previous studies have shown that pre-weaning socialization offers behavioral benefits and may enhance early post-weaning feed intake and reduce stress [5], our results may reflect the acute effects of weaning stress in both treatment groups [1]. The high stress levels at weaning time may override the more subtle modulatory effects of early-life socialization, as concluded by Blavi et al. [16] and Moscovice et al. [17]. The physiological impact of socialization has been investigated in many studies [9], with some reports indicating a small increase or decrease in stress hormones such as cortisol in socialized animals, as shown by Ji et al. [8], who reported apparently higher cortisol levels for continuously socialized piglets vs. controls and significantly higher vs. the intermittently socialized animals. Other authors showed reduced cortisol levels in previously socialized animals, as reported by Salazar et al. [5], who found no differences in basal salivary cortisol between treatments before socializing animals (on day 7 or 14 of life) but observed a significant post-weaning increase in cortisol in non-socialized piglets. Therefore, considering that saliva samples were collected at 10 a.m., to avoid the morning peak in cortisol secretion and reduce circadian variation [13], this absence of significant differences in our study does not necessarily imply a lack of treatment effect but may instead indicate that these markers are saturated (they have reached its maximum response even if the stimulus continues to rise) and may not be sufficiently discriminating to detect subtle treatment differences in stress between CTRL and SOC piglets when tested under high-stress conditions. This aligns with the study in commercial breeds by Van Kerschaver et al. [4], who also described that HPA-related markers at weaning often show limited sensitivity to management practices due to the intensity of the stressor. Therefore, our results may suggest that Iberian piglets respond similarly to commercial breeds in terms of acute HPA activation at weaning after a socialization strategy. Moreover, the high individual variability in salivary biomarkers under acute stress, as described by Moscovice et al. [17], could have further masked potential differences induced by pre-weaning suggesting that the practical application of these findings could extend beyond this autochthonous breed.

An alternative explanation could be that SOC piglets would have experienced higher pre-weaning levels of stress caused by short-lived aggression just after mixing, as reported by Salazar et al. [5], thus possibly affecting post-weaning stress biomarker values. However, this hypothesis requires further investigation regarding behavioral observations.

In contrast to cortisol and cortisone, CgA, a marker of sympathoadrenal system activation [11], showed more discriminating results. In our study, on pwD7 SOC piglets showed a marked reduction in CgA levels in comparison with CTRL piglets. To our knowledge, no previous studies have evaluated salivary CgA in socialized piglets whether in Iberian or commercial breeds. However, its general role as a neuroendocrine stress marker has been demonstrated in response to acute stressors such as castration [18]. Elevated CgA has been associated with increased cortisol levels and social stress [18]. This reduction may reflect a lower activation of the sympatho-adreno-medullary system [2], which may indicate a potential beneficial effect of pre-weaning socialization on the physiological adaptation process during the post-weaning period. The fact that CgA showed significant differences while cortisol did not may suggest that sympathoadrenal activation was more sensitive to the effects of pre-weaning socialization than HPA axis activation, or that the single-point sampling strategy was more appropriate for detecting CgA changes. This differential response highlights the value of assessing multiple stress pathways simultaneously.

On pwD7, while classical stress hormones (cortisol and cortisone) did not reflect any treatment effect, oxytocin showed a trend to increase in the SOC group, in contrast to the trend for an increase that was observed in CTRL at weaning. Oxytocin levels are commonly linked to social bonding and positive emotional regulation [11,19]. However, it is important to note that some authors consider that salivary oxytocin is not a reliable indicator of emotional well-being in pigs [17]. Therefore, our findings regarding oxytocin should be interpreted with caution. Nevertheless, they may suggest improved social interaction in SOC piglets during the first week post-weaning, as supported by Valros et al. [20], who reported that oxytocin levels tended to be higher in pigs without tail-biting which suggests a better emotional status.

Butyryl-cholinesterase (BChE), a major component of total esterase activity (TEA), is an enzyme synthesized primarily in the liver that plays a key role in the enzymatic response to acute stress [21,22], with activity typically increasing during stress conditions along with prolonged states of distress or discomfort [22]. While BChE activity may transiently increase under acute sympathetic activation, prolonged stress or reduced feed intake can result in lower circulating levels due to impaired hepatic synthesis or altered metabolic state [21]. In our study, although no significant treatment effects were observed for BChE on pwD7, we did find a lower enzymatic activity in females than in males. This sex difference might be related to hormonal influences on hepatic enzyme activity, although additional research is needed to confirm this hypothesis.

Another stress related biomarker, such as Paraoxonase-1 (PON-1) was measured but showed no significant differences between treatment groups. PON-1 is an antioxidant enzyme associated with HDL that protects against oxidative stress and lipid peroxidation [23]. Since PON-1 activity was not affected by treatment in our study, the lack of difference suggests that oxidative stress status could be similar between groups at pwD7, despite the differences observed in other stress and inflammatory markers.

### 4.2. Inflammatory and Immune Markers

The evaluation of inflammatory and immune status through salivary ADA and serum acute-phase proteins provides insights into how pre-weaning socialization affects the inflammatory response to weaning stress. Interestingly, our data show that ADA, a marker indicative of immune activation in pigs [24] was significantly reduced in the SOC group at weaning. Since increased ADA activity has been associated with inflammatory responses to stress [22] this reduction may suggest that pre-weaning socialization attenuated the immunologic activation arising from the acute stress of weaning. This reduction persisted on pwD7, where ADA showed a trend to remain lower in SOC piglets. Biologically, the reduction in ADA activity observed in socialized piglets may indicate a lower degree of T-cell activation or lymphocyte turnover in response to stress [24], thus suggesting that pre-weaning socialization starting at 15 days of life may have mitigated an excessive activation of the immune system in response to weaning stress, hence potentially preventing an excessively inflammatory status.

The acute-phase proteins CRP and haptoglobin are commonly used as indicators of systemic inflammation, that can be related to stress in pigs [2,25]. As stated by various authors, these biomarkers typically increase in response to stressors such as weaning, regrouping or dietary changes, reflecting the inflammatory impact of such challenges [12]. In the present study, CRP levels were not significantly affected by treatment or sex. However, haptoglobin concentrations were markedly lower in SOC piglets vs. CTRL piglets. This result supports the hypothesis that pre-weaning socialization may later attenuate stress and the haptoglobin response during the critical post-weaning period. Although specific studies on haptoglobin levels in socialized piglets are scarce, similar and comparable reductions in inflammatory markers have been reported in commercial breeds, where environmental enrichment and increased social contact during early life led to reduced acute-phase protein responses to subsequent stressors [26]. Thus, the consistency between ADA and haptoglobin results is noteworthy. Both markers, despite being measured in different biological matrices (saliva and serum, respectively) and reflecting different aspects of immune/inflammatory activation, showed lower values in socialized piglets. This convergence of findings strengthens the hypothesis that pre-weaning socialization could attenuate the stress syndrome response to weaning, experiencing less reactive immune system activation and a more adaptive inflammatory response during the post weaning period.

Regarding Immunoglobulin G (IgG), it was also measured in order to reflect the humoral immune status but showed no significant differences between treatment groups. IgG concentration was similar between treatments, and that could be indicating that pre-weaning socialization did not compromise or enhance systemic antibody levels at this sampling point. These findings may suggest that the benefits of socialization on stress did not extend to all aspects of immune function, or that a longer observation period might be necessary to detect changes in these parameters.

### 4.3. Metabolic Parameters

In the present study, several lipid metabolism parameters showed clear treatment effects. Interestingly, SOC piglets exhibited significantly higher concentrations of HDL, LDL, and total cholesterol compared to CTRL animals. Previous research in commercial pig breeds has shown that weaning stress can disrupt lipid metabolic pathways, impair lipoprotein synthesis, and negatively affect energy balance and growth. Specifically, these studies have reported decreased levels of HDL and LDL following weaning stress, associated with reduced hepatic lipoprotein synthesis and altered lipid metabolism [27,28]. Therefore, this maintenance of lipid homeostasis appears to be independent of breed, since our findings are consistent with those studies and may also reflect that SOC piglets maintained a more stable lipidic profile compared to CTRL animals, possibly due to a lower stress-induced catabolism. This similarity indicates that, although Iberian pigs possess distinct lipid deposition patterns, their short-term metabolic responses to socialization follow trends comparable to those of fast-growing lines.

We also observed that SOC piglets had significantly higher blood glucose concentrations compared to CTRL piglets, which may suggest enhanced energy availability. In fact, weaning has been reported to reduce blood glucose levels, potentially limiting growth due to decreased energy reserves and reduced feed intake [29,30]. The higher glucose concentrations observed in the SOC group are consistent with the study from Van Kerschaver et al. [4], in which pre-weaning socialized piglets showed higher concentration of glucose in blood than the Control group at day 2 post-weaning.

Taken together, the higher levels of lipoproteins (HDL, LDL, cholesterol) and glucose in socialized piglets may suggest a more favorable energy metabolism profile at pwD7. This could reflect that socialized piglets were calmer and faced less distress during the post-weaning period, as previous salivary and serum biomarkers showed, possibly resuming feed intake earlier as supported by the enhanced growth observed in these animals during the early post-weaning period (D30–D45).

Regarding nitrogen metabolism, urea (a byproduct of protein catabolism) serves as a useful indicator of amino acid and protein utilization efficiency [31,32]. Elevated blood urea nitrogen (BUN) has been linked to inefficient protein synthesis and increased muscle breakdown [33]. According to our findings, SOC piglets exhibited significantly lower urea levels, suggesting reduced protein catabolism or improved amino acid utilization. These lower urea levels in SOC piglets are also consistent with the hypothesis of improved metabolic efficiency and may be related to the observed higher growth rates during the D30–D45 period in this treatment group. In addition, the significantly lower serum urea levels observed in males than in females may be associated with more efficient protein metabolism, possibly due to hormonal influences. Although our piglets were very young (37 days old), to our knowledge, no studies have reported sex-related effects at such an early age in serum urea levels. Only a few authors, such as Le Floc’h et al. [34], have described similar patterns in older animals, finding lower plasma urea levels in intact males than in castrated males. Therefore, our results may provide useful preliminary information for future studies investigating nitrogen metabolism in piglets. In contrast, no significant treatment effects were found for triglycerides, creatinine, total proteins, or lactate. Although females’ piglets showed a trend for higher creatinine levels that warrants additional investigation.

### 4.4. Growth Performance

Body weight and ADG reflect the nutritional status and the physiological impact of stress [35]. In our study, no significant differences in body weight were observed between treatment groups at any time point (Figure 2), therefore the changes observed in average daily gains are not due to initial weight, but probably to an effect of stress in the different periods. Previous studies have reported contradictory results regarding the effect of pre-weaning socialization on growth. Some studies suggest that this procedure improves exploratory behavior and reduces aggressive interactions, indirectly favoring post-weaning performance [5], but other authors report either reduced weaning weights in socialized piglets [3] or no significant effects during the first two post-weaning weeks [4]. Kanaan et al. [36] found no effect on BW gain, which is in line with our findings. Such discrepancies across studies could stem from differences in experimental design, breeds, or environmental conditions.

Regarding ADG, SOC piglets showed significantly higher growth between days 30 and 45 post-weaning (Figure 3). This may indicate reduced stress, and thus improved adaptation, during the early post-weaning period. Our findings are consistent with the study from Van Kerschaver et al. [4], who observed higher ADG in socialized piglets during the early post-weaning period. These results contrast with some studies, such as Salazar et al. [5], in which no significant differences in post-weaning growth were reported between socialized and control animals.

Interestingly, from day 45 to day 60 of life, SOC piglets exhibited a reduced growth rate compared to CTRL animals (0.30 vs. 0.40 kg/day, *p* = 0.049). This pattern of differential growth may be explained by a compensatory growth in the CTRL group, possibly due to a delayed adaptation to the earlier stressors, supported by the highest values observed in the biomarkers of stress and inflammation (CgA and haptoglobin) on day 7 post-weaning. This possible compensatory growth could also highlight the inherent rusticity and resilience of the Iberian pig breed [6]. Moreover, no significant differences in ADG were found during the pre-weaning period, suggesting that the effects of socialization on growth performance may show up more clearly under the challenging conditions of the post-weaning period. It is important to note that, when considering the entire post-weaning period (D30–D60), no significant differences in ADG were observed between treatments (0.26 vs. 0.27 kg/day for SOC vs. CTRL, *p* = 0.742), indicating that overall post-weaning growth performance was similar despite temporal variation in growth rates. However, it is necessary to highlight that in the most critical post-weaning period (D30–45), SOC piglets presented higher ADG than the CTRL group. This suggests that during this period, the SOC group experienced less impact from post-weaning stress (Figure 3).

## 5. Conclusions

Even though all piglets experienced stress during weaning, the convergent evidence from multiple biomarkers—reduced salivary ADA and CgA, lower serum haptoglobin, and improved metabolic profiles (higher lipoproteins, glucose, and lower urea)—may indicate attenuated inflammatory and stress responses along with enhanced energy homeostasis in socialized piglets during the critical post-weaning period. The improved ADG observed between D30 and D45 further supports more effective physiological adaptation, despite subsequent compensatory growth equalizing final body weights between groups.

Although this multi-biomarker approach may provide an integrated physiological perspective, future studies should refine marker selection and incorporate behavioral indicators to more comprehensively characterize the benefits of socialization.

In addition, Iberian piglets appeared to respond to socialization in a similar manner to commercial breeds, suggesting that the practical application of these findings could extend beyond this autochthonous breed. Nevertheless, future research conducted at the moment of socialization-including both stress biomarkers and behavioral assessments, as well as the evaluation of different socialization strategies-remains necessary.

## Figures and Tables

**Table 1:** Ingredients and nutrient levels of the basal diet (%).

Ingredients	Content	Nutrient Levels	Content
Barley	21.00	Crude protein	16.99
Wheat	16.00	Crude fat	4.76
Corn	13.50	Crude fiber	4.14
Soybean meal	11.80	Lysine	1.25
Yeast products	8.00	Methionine	0.50
Soybean concentrate	7.00	Sodium	0.32
Monocalcium phosphate	6.50	Calcium	0.40
Corn flakes	3.00	Phosphorus	0.50
Barley flakes	2.50	Crude ashes	4.25
Sodium chloride	1.30		
Calcium carbonate	1.10		
Sodium salts	1.00		
Dehydrated seaweed (*Chlorella vulgaris*)	0.90		
*Aspergillus* by-products	0.85		
Vegetable protein	0.80		
Sweet whey	0.75		
Re-fatted whey	0.70		
Beet pulp	0.60		
Soybean oil	0.58		
*Saccharomyces cerevisiae*	0.53		
L-Lysine	0.37		
DL-Methionine	0.18		
L-Threonine	0.22		
L-Tryptophan	0.07		
L-Valine	0.09		
Vitamin-Mineral premix ^1^	0.50		
Preservatives (E237)	0.16		

^1^ Vitamin premix provided per kilogram of diet: Vitamin A, 10002 IU; Vitamin D3, 2004 IU; Vitamin E, 50 mg; Vitamin K3, 5 mg; Vitamin B1 monohydrate, 4. 35 mg; Vitamin B2, 5 mg; Vitamin B6 Pyridoxine hydrochloride, 10 mg; Vitamin B12, 36 mg; Biotin, 0.50 mg; Niacinamide, 27.50 mg; D-Calcium pantothenate, 15.40 mg; Folic acid, 2.5 mg; Choline chloride, 280.30 mg; Betaine anhydrous, 252 mg. Mineral premix provided per kilogram of diet: Iron, 80 mg; Iodine, 2 mg; Copper, 99.97 mg; Manganese, 30 mg; Zinc, 80 mg; Selenium, 0.2 mg.

**Table 2:** Salivary Cortisol, Cortisone, Oxytocin, Chromogranin A (CgA), Alpha-Amylase (sAA) and Adenosine Deaminase (ADA) in Control (CTRL) and Socialized (SOC)); Female (F) and Male (M) piglets at weaning and on day 7 post-weaning.

Saliva Biomarkers at Weaning and on Day 7 Post-Weaning						
Variables	Group		Sex		*p* Value	
	CTRL	SOC	F	M	Group	Sex
At weaning						
Cortisol D0 (µg/dL)	0.26 ± 0.04	0.26 ± 0.04	0.29 ± 0.04	0.23 ± 0.04	0.961	0.263
Cortisone D0 (ng/mL)	2.88 ± 0.37	2.80 ± 0.36	2.93 ± 0.38	2.75 ± 0.35	0.879	0.725
Oxytocin D0 (pg/mL)	5845.15 ± 459.01 †	4688.75 ± 445.31 †	5404.36 ± 470.72	5129.53 ± 436.17	0.080	0.674
CgA D0 (ng/mL)	641.51 ± 77.89	692.21 ± 75.57	663.83 ± 79.88	669.88 ± 74.02	0.645	0.956
sAA D0 (U/L)	6688.66 ± 2183.37	8566.50 ± 2118.20	8481.67 ± 2239.08	6773.50 ± 2074.74	0.543	0.582
ADA D0 (U/L)	5723.08 ± 674.42 ^a^	2522.03 ± 654.29 ^b^	4437.46 ± 691.63	3807.66 ± 640.87	0.002	0.512
Day 7 post-weaning						
Cortisol D7 (µg/dL)	0.10 ± 0.04	0.12 ± 0.04	0.13 ± 0.04	0.09 ± 0.04	0.661	0.469
Cortisone D7 (ng/mL)	2.93 ± 0.44	1.88 ± 0.44	2.12 ± 0.47	2.69 ± 0.43	0.102	0.381
Oxytocin D7 (pg/mL)	4974.40 ± 475.04 †	6125.50 ± 472.98 †	5435.60 ± 501.11	5664.31 ± 457.11	0.094	0.741
CgA D7 (ng/mL)	787.80 ± 69.64 ^a^	518.38 ± 69.34 ^b^	732.79 ± 73.47	573.38 ± 67.01	0.009	0.122
sAA D7 (U/L)	7900.96 ± 1531.57	5774.75 ± 1524.92	6313.43 ± 1615.63	7362.28 ± 1473.75	0.332	0.639
ADA D7 (U/L)	5478.00 ± 672.74 †	3789.83 ± 669.82 †	4928.38 ± 709.66	4339.45 ± 647.34	0.084	0.549

Within effect, means with different superscript letters between columns differ significantly (*p* < 0.05); † indicates a trend (0.05 ≤ *p* < 0.10). Data are shown as LSMEANS (Least Square Means) ± SE (Standard Error) and *p* values. D0 = biomarker measured at weaning; D7 = biomarker measured 7 days post-weaning.

**Table 3:** Serum Butyryl-cholinesterase (BChE), C-Reactive Protein (CRP), Haptoglobin, Paraoxonase-1 (PON-1), and Immunoglobulin G in Control (CTRL) and Socialized (SOC) Female (F) and Male (M) piglets on day 7 post-weaning.

Serum Parameters on Day 7 Post-Weaning						
Variables	Group		Sex		*p* Value	
	CTRL	SOC	F	M	Group	Sex
BChE (µmol mL/minute)	0.45 ± 0.01	0.46 ± 0.01	0.41 ± 0.01 ^a^	0.50 ± 0.01 ^b^	0.311	<0.0001
CRP (mg/L)	56.91 ± 5.91	51.70 ± 5.88	56.19 ± 6.23	52.42 ± 5.68	0.537	0.662
Haptoglobin (g/L)	1.54 ± 0.14 ^a^	1.12 ± 0.14 ^b^	1.48 ± 0.15	1.18 ± 0.13	0.036	0.132
PON-1 (U/L)	47.99 ± 2.58	51.21 ± 2.57	49.14 ± 2.73	50.06 ± 2.49	0.383	0.807
Immunoglobulin G (mg/L)	4.69 ± 0.27	4.37 ± 0.27	4.54 ± 0.29	4.53 ± 0.26	0.405	0.972

Within effect, means with different superscript letters between columns differ significantly (*p* < 0.05). Data are shown as LSMEANS (Least Square Means) ± SE (Standard Error) and *p* values.

**Table 4:** Serum parameters in Control (CTRL) and Socialized (SOC) Female (F) and Male (M) piglets on day 7 post-weaning.

Serum Parameters on Day 7 Post-Weaning						
Variables	Group		Sex		*p* Value	
	CTRL	SOC	F	M	Group	Sex
HDL * (mg/dL)	32.56 ± 1.60 ^a^	45.71 ± 1.54 ^b^	41.26 ± 1.66 †	37.02 ± 1.48 †	<0.0001	0.064
LDL * (mg/dL)	25.67 ± 1.70 ^a^	37.26 ± 1.64 ^b^	31.59 ± 1.77	31.34 ± 1.57	<0.0001	0.918
Total Cholesterol (mg/dL)	76.33 ± 3.76 ^a^	102.77 ± 3.62 ^b^	92.69 ± 3.90	86.41 ± 3.47	<0.0001	0.236
Triglycerides (mg/dL)	52.40 ± 3.72	49.72 ± 3.58	53.79 ± 3.85	48.34 ± 3.43	0.606	0.297
Creatinine (mg/dL)	0.46 ± 0.02	0.45 ± 0.01	0.47 ± 0.02 †	0.43 ± 0.01 †	0.607	0.057
Urea (mg/dL)	19.10 ± 1.04 ^a^	12.06 ± 1.00 ^b^	18.90 ± 1.08 ^a^	12.26 ± 0.96 ^b^	<0.0001	<0.0001
Total Proteins (g/L)	55.13 ± 0.66	54.51 ± 0.63	55.34 ± 0.68	54.30 ± 0.61	0.505	0.264
Glucose (mg/dL)	101.85 ± 2.91 ^a^	119.13 ± 2.81 ^b^	108.52 ± 3.02	112.47 ± 2.69	0.0001	0.335
Lactate (mg/dL)	64.00 ± 3.41	57.92 ± 3.28	61.57 ± 3.54	60.35 ± 3.15	0.206	0.798

Within effect, means with different superscript letters between columns differ significantly (*p* < 0.05); † indicates a trend (0.05 ≤ *p* < 0.10). Data are shown as LSMEANS (Least Square Means) ± SE (Standard Error) and *p* values. * HDL (High Density Lipoproteins), LDL (Low Density Lipoproteins).

## Data Availability

The data supporting the findings of this study are available from the corresponding author upon reasonable request. Data is included within the article.

## Abbreviations

The following abbreviations are used in this manuscript:

ADA	Adenosine Deaminase
ADG	Average Daily Gain
BChE	Butyryl-cholinesterase
BW	Body Weight
BUN	Blood Urea Nitrogen
CgA	Chromogranin A
Cort	Cortisol
Crts	Cortisone
CRP	C-Reactive Protein
CTRL	Control group
D30	Day 30 of age
D45	Day 45 of age
D60	Day 60 of age
F	Female
GLM	Generalized Linear Model
HDL	High Density Lipoprotein
Hp	Haptoglobin
HPA	Hypothalamic–pituitary–adrenal
IgG	Immunoglobulin G
LD0	Lactation day 0 (birth)
LD15	Lactation day 15
LD30	Weaning
LDL	Low Density Lipoprotein
M	Male
n	Number of observations (litters/animals) per group
OT	Oxytocin
PON-1	Paraoxonase 1
pwD0	Weaning
pwD7	Post-weaning day 7
pwD15	Post-weaning day 15
pwD30	Post-weaning day 30
sAA	Salivary Alpha-Amylase
SAM	Sympatho-adreno-medullary
SOC	Socialization group
TEA	Total Esterase Activity
ZnO	Zinc Oxide
